# Supplementary material for: Levodopa Rescues Retinal Function in the Transgenic A53T Alpha-Synuclein Model of Parkinson’s Disease
Source: Biomedicines. 2024 Jan 8;12(1):130. doi: 10.3390/biomedicines12010130 (PMC10813165; doi:10.3390/biomedicines12010130)
Supplement: Supplementary file 1 [file biomedicines-12-00130-s001.zip › biomedicines-2710424-supplementary.pdf]

Supplementary Material

Levodopa Rescues Retinal Function in the Transgenic A53T Alpha-synuclein Model of Parkinson's Disease

Katie K.N. Tran[1], *Vickie H.Y. Wong*[1], *Kirstan A. Vessey*[2], *David I. Finkelstein*[3], *Bang V. Bui*[1], *Christine T.O. Nguyen*[1*]

[1] Department of Optometry and Vision Sciences, The University of Melbourne, Parkville, 3010, Victoria, Australia.

[2] Department of Anatomy and Physiology, The University of Melbourne, Parkville, 3010, Victoria, Australia.

[3] The Florey Institute of Neuroscience and Mental Health, The University of Melbourne, Parkville, 3010, Victoria, Australia.

***Correspondence**: Christine T.O. Nguyen: christine.nguyen@unimelb.edu.au

Supplementary Figure

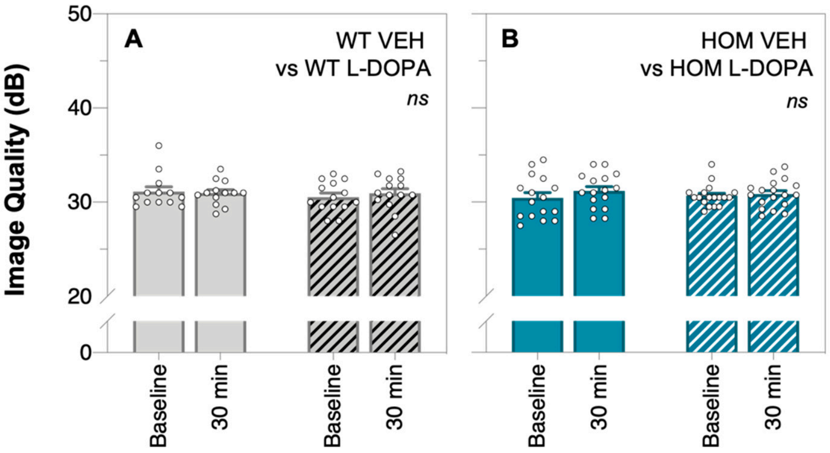

Supplementary Figure S1. OCT image quality over time before and after drug treatment. Optical coherence tomography (OCT) image quality as measured in decibels (dB) of wildtype (WT, grey **A**) animals (plain, VEH treated, *n* = 13; black-striped, L-DOPA treated, *n* = 14) and A53T homozygous (HOM, teal **B**) mice (plain, VEH-treated, *n* = 15; white-striped, L-DOPA-treated, *n* = 18) before and after levodopa (L-DOPA) or vehicle (VEH) treatment. Image quality remained stable over time and robust to drug treatment. All data shown, mean ± SEM
